# Discovery and preclinical development of [^18^F]ACI‐19626, a first‐in‐class TDP‐43 PET tracer

**DOI:** 10.1002/alz.083411

**Published:** 2025-01-09

**Authors:** Tamara Seredenina

**Affiliations:** ^1^ AC Immune, Lausanne, Vaud Switzerland

## Abstract

**Background:**

Aggregated TDP‐43 is found as the main component of pathological inclusions in amyotrophic lateral sclerosis (ALS), limbic‐predominant age‐related TDP‐43 encephalopathy (LATE), in 50% of patients with frontotemporal dementia (FTD), and is present as co‐pathology in other neurodegenerative diseases, including Alzheimer’s disease (AD). Biofluid or imaging biomarkers of TDP‐43 pathology are currently unavailable. Direct detection of TDP‐43 aggregates holds promise for unraveling the pathobiology of disease and for enabling precision medicine approach via improved diagnosis, patient stratification and assessment of therapeutic efficacy in clinical trials in AD and related diseases.

**Methods:**

Using our Morphomer® small molecule library of brain‐penetrant and beta sheet binding compounds, initial hits were identified using patients’ brain material. Radiobinding and autoradiography assays were used to assess binding affinity and target engagement on brain samples from cases with TDP‐43 proteinopathies, and selectivity over other aggregation‐prone proteins on AD and Parkinson’s disease (PD) tissue. The pharmacokinetics in the brain was studied in mice, followed by evaluation in cynomolgus monkeys to assess brain uptake, distribution and washout for selected 18F‐radiolabeled compounds.

**Results:**

Screening of >500 compounds led to identification of several distinct chemical series that bound pathological TDP‐43 derived from FTLD‐TDP brain samples with nanomolar affinity. Rational drug design and medicinal chemistry optimization enabled the discovery of the TDP‐43 tracer, ACI‐19626. This compound has high affinity binding (low nM Kd) for human brain‐derived aggregated TDP‐43 and can differentiate FTLD‐TDP type A and B patient samples from age‐matched controls. ACI‐19626 displays selectivity for TDP‐43 over Abeta, Tau and alpha‐synuclein aggregates, and no binding to common off‐target proteins, including monoamine oxidase A and B. In monkeys, [^18^F]ACI‐19626 has a rapid brain uptake and fast and complete washout, a profile suitable for brain PET imaging.

**Conclusions:**

[^18^F]ACI‐19626 was identified as a potential first‐in‐class TDP‐43 PET tracer and selected for evaluation in a First‐in‐Human study based on its excellent affinity, selectivity and pharmacokinetic properties. [^18^F]ACI‐19626 has the potential to detect and monitor the progression of TDP‐43 pathology in brains of living patients with TDP‐43 proteinopathies such as ALS, FTLD‐TDP, AD and LATE, enabling precision medicine approach and combination therapies.

